# Interaction of [Rh_2_(O_2_CCH_3_)_4_(H_2_O)_2_] and [Rh_2_(O_2_CCH(OH)Ph)_2_(phen)_2_(H_2_O)_2_](O_2_C-CH(OH)Ph)_2_ With Sulfhydryl Compounds and Ceruloplasmin

**DOI:** 10.1155/MBD.2000.201

**Published:** 2000

**Authors:** Piotr Jakimowicz, Lucja Ostropolska, Florian P. Pruchnik

**Affiliations:** Faculty of Chemistry University of Wroclaw 14 Joliot-Curie Street Wroclaw 50-383 Poland

## Abstract

The interaction of binuclear rhodium(II) complexes [Rh_2_(OOCCH_3_)_4_(H_2_O)_2_], [Rh_2_{OOCCH(OH)Ph}_2_(phen)_2_(H_2_O)_2_] {OOCCH(OH)Ph}_2_, [Rh_2_(OOCCH_3_)_2_(bpy)_2_(H_2_O)_2_](OOCCH_3_)_2_ and [Rh_2_Cl_2_(OOCMe)_2_(bpy)_2_](3H_2_O) with ceruloplasmin, cysteine, glutathione and coenzyme A have been investigated using. UV-Vis and CD spectroscopies. The complexes containing phen or bpy at pH = 7.4 and 4.0 are readily reduced with sulfhydryl compounds, while rhodium(II) acetate is relatively stable in these conditions. Complex [Rh_2_{OOCCH(OH)Ph}_2_(phen)_2_(H_2_O)_2_] strongly changes structure of ceruloplasmin leading to the decrease of of α-helix content and loss of oxidase activity.


### INTERACTION OF $[Rh_2(O_2CCH_3)_4(H_2O)_2]$ AND $[Rh_2(O_2CCH(OH)Ph)_2(phen)_2(H_2O)_2](O_2C\text{-}CH(OH)Ph)_2$ WITH SULFHYDRYL COMPOUNDS AND CERULOPLASMIN

Piotr Jakimowicz, Lucja Ostropolska and Florian P. Pruchnik*

Faculty of Chemistry, University of Wroclaw, 14 Joliot-Curie Street, 50-383 Wroclaw, Poland
<pruchnik@wchuwr.chem.uni.wroc.pl>

### ABSTRACT

The interaction of binuclear rhodium(II) complexes $[Rh_2(OOCCH_3)_4(H_2O)_2]$, $[Rh_2\{OOCCH(OH)Ph\}_2(phen)_2(H_2O)_2]\{OOCCH(OH)Ph\}_2$, $[Rh_2(OOCCH_3)_2(bpy)_2(H_2O)_2](OOCCH_3)_2$ and $[Rh_2Cl_2(OOCMe)_2(bpy)_2](3H_2O)$ with ceruloplasmin, cysteine, glutathione and coenzyme A have been investigated using UV-Vis and CD spectroscopies. The complexes containing phen or bpy at pH = 7.4 and 4.0 are readily reduced with sulfhydryl compounds, while rhodium(II) acetate is relatively stable in these conditions. Complex $[Rh_2\{OOCCH(OH)Ph\}_2(phen)_2(H_2O)_2]$ strongly changes structure of ceruloplasmin leading to the decrease of α-helix content and loss of oxidase activity.

### INTRODUCTION

Dimeric rhodium(II) complexes have attracted considerable interest due to their interesting structure and reactivity [1-10], catalytic properties [1-3,5,11-13] and anticancer activity [1-3,5,10,14-19]. The structure, reactivity, catalytic properties and cytostatic activity of $[Rh_2X_2(OOCR)_2(N\text{-}N)_2]$ and $[Rh_2(OOCR)_2(N\text{-}N)_2(H_2O)_2]^{2+}$ complexes (X = Cl, Br, I; R = H, Me, PhCHOH; N-N = 2,2'-bipyridine, 1,10-phenanthroline and their derivatives) have been investigated [4-13]. Rhodium(II) carboxylato complexes belong to the most promising platinum metals compounds having anticancer and biological activity [14-21]. These results have prompted investigations into the chemical properties and biological effects of rhodium(II) complexes [14-16, 20-26]. It has been found [27-28] that $[Rh_2(OOCR)_2(N\text{-}N)_2(H_2O)_2]^{2+}$ complexes show cytostatic activity against human oral carcinoma KB cell line in vitro. They are also cytostatic agents against synchronously cultivated cultures of green algae *chlorella vulgaris* [29-30]. The compounds $[Rh_2(OOCR)_2(N\text{-}N)_2(H_2O)_2]^{2+}$ and $[Rh_2Cl_2(OOCR)_2(N\text{-}N)_2]$ are effective antibacterial agents [31-33].

Thus the investigation of interaction of Rh(II) complexes with compounds playing crucial role in biological processes is interesting. Glutathione (GSH) is a naturally occurring tripeptide with the sequence γ-L-glutamyl-L-cysteinyl-glycine. GSH is generally the most abundant intracellular nonprotein thiol. For example, in human erythrocytes, GSH is present typically at the 2 to 3 mM level. GSH is taking part, directly or in the presence of enzymes, in neutralization processes of radicals and molecules potentially noxious for living organisms. Its role is especially important in preventing autooxidation of cells during respiration processes. Ceruloplasmin is an enzyme playing an important role. It belongs to the $\alpha_2$-globulin part of blood plasma. Its physiological function is not entirely explained. To the relatively well recognized functions belong: copper transport, ferro oxidase, amino oxidase and anti-oxidant activity. Human ceruloplasmin contains six tightly bound copper ions; however it can bind two additional labile $Cu^{2+}$ ions [34-39]. Strongly bound ions are classified as three types of $Cu^{2+}$ on the basis of their spectral properties. There are three $Cu^{2+}$ of type 1, one $Cu^{2+}$ of type 2 and a dinuclear center with type 3 $Cu^{2+}$ ions. Type 1 coppers have characteristic electronic and EPR spectra. They show an intense band at 610 nm ($\varepsilon \approx 10000\ M^{-1}cm^{-1}$) responsible for blue color of ceruloplasmin. In comparison, the extinction coefficient of the $Cu(H_2O)_6^{2+}$ complex is *ca* 10 $M^{-1}cm^{-1}$. They also give EPR signal with narrow hyperfine splitting from copper ($A_{||} \approx 7$ mT). The electronic and EPR spectra of type 2 $Cu^{2+}$ are similar to those of the low molecular copper complexes ($A_{||} \approx 17$ mT). The two coppers of type 3 interact with one $Cu^{2+}$ ion of type 2 and form a trinuclear cluster. Two $Cu^{2+}$ ions of type 3 are diamagnetic because of a strong antiferromagnetic interaction. They are bridged by the oxygen atom and are responsible for a shoulder at 330 nm in the absorption spectra. However, describing of the trinuclear center as the combination of a type 2 and a type 3 copper center is unfortunate because the distribution of the ligands and catalytic properties differ from those of type 2 and 3 centers. Thus, the trinuclear copper center should be recognized as a separate type of copper cluster [34-36]. An increased level of ceruloplasmin is observed during infectious diseases and cancer and GSH can form metal complexes with potential antitumor activity.

Therefore it seemed interesting to investigate the interaction of $[Rh_2(OOCR)_4]$ and $[Rh_2(OOCR)_2(N\text{-}N)_2(H_2O)_2]^{2+}$ complexes with proteins occurring in human plasma, with some aminoacids and compounds containing sulfhydryl groups. In this paper, we describe the interaction of $[Rh_2(OOCCH_3)_4(H_2O)_2]$, $[Rh_2(OOCCH_3)_2(bpy)_2(H_2O)_2](OOCCH_3)_2$, $[Rh_2Cl_2(OOCCH_3)_2(bpy)_2](3H_2O)$ and $[Rh_2(OOCCH(OH)Ph)_2(phen)_2(H_2O)_2](OOCCH(OH)Ph)_2$ with ceruloplasmin and L-methionine, L-cysteine, L-histidine, glutathione and coenzyme A.

### MATERIALS AND METHODS

$[Rh_2(OOCCH_3)_4(H_2O)_2]$ [40-41] (**1**), $Na_4[Rh_2(CO_3)_4](2.5H_2O)$ [42], $[Rh_2(OOCCH_3)_2(bpy)_2(H_2O)_2](OOCCH_3)_2$ [26] (**2**), $[Rh_2Cl_2(OOCCH_3)_2(bpy)_2](3H_2O$ [9] (**3**) and $[Rh_2(OOCCH(OH)Ph)_2\text{-}(phen)_2(H_2O)_2](OOCCH(OH)Ph)_2$ [29] (**4**) were prepared by literature methods. The purity of all the complexes was controlled by elemental analysis and IR spectroscopy. L-methionine, L-cysteine, L-histidine, coenzyme A and glutathione were obtained from Aldrich and used without further purification. Ceruloplasmin obtained from Plant of Serum and Vaccines (Warsaw) was purified by dialysis before reactions. It exhibited an A610/A278 absorbance ratio of 0.039. All measurements were carried out in 0.01 M $NaOOCH_3/HOOCCH_3$ (pH = 4.0) and 0.01 M $NaH_2PO_4$/ $Na_2HPO_4$ (pH = 7.4) buffers. The enzyme activity of the protein was determined by a literature method involving the oxidation of *p*-phenylenediamine (PPD) [43]. Electronic spectra were recorded on a Cary 5 and a Beckman 7500 spectrometer connected with Hi-Tech stopped flow equipment, CD spectra, on a Jasco J600 spectrometer and IR spectra, on an IFS 113v Bruker.

### RESULTS AND DISCUSSION

The interaction of rhodium complexes with L-methionine, L-cysteine, L-histidine, coenzyme A and glutathione has been investigated using absorption electronic and CD spectroscopy. Reactions were carried out in water at pH 4.0 and 7.4 in acetate and phosphate buffers, respectively, at room temperature under nitrogen.

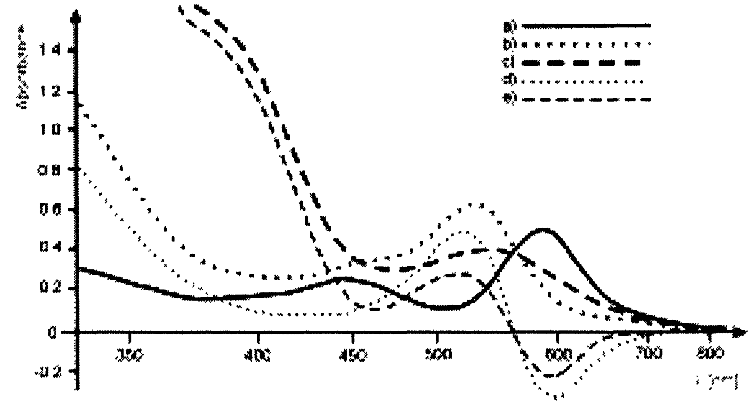

Figure 1. Absorption spectra: a) complex **1**; b) **1**+histidine after 1 min; c) **1**+histidine after 24 hours; d) difference spectrum of (**1**+histidine) / (histidine, **1**) after 1 min; e) difference spectrum of (**1**+histidine) / (histidine, **1**) after 24 hours; in 0.01M phosphate buffer, pH= 7.4, [**1**]=[$3\cdot10^{-3}$ M] and [histidine]=[$3\cdot10^{-2}$ M]

In the electronic spectra of complexes **2**, **3** and **4** in the visible region, two bands are observed. Band I occurs at 560 - 570 nm, about 25 nm lower compared with that for $Rh_2(OOCR)_4(H_2O)_2]$ compounds and corresponds to the $\pi^*(Rh_2)\rightarrow\sigma^*(Rh_2)$ transition. This blue shift is caused by the stabilization of $\pi^*(Rh_2)$ levels in $[Rh_2X_2(OOCR)_2(N\text{-}N)_2]$ and $[Rh_2(OOCR)_2(N\text{-}N)_2(H_2O)_2]^{2+}$ complexes due to the interaction of the orbitals of the $Rh_2$ core with $\pi^*$ orbitals of nitrogen ligands. Thus they have a lower energy than the $\pi^*(Rh_2)(5e_g)$ orbitals in $[Rh_2(OOCR)_4(H_2O)_2]$ complexes. The intense band II at 410 - 420 nm was assigned to the allowed charge-transfer transition $\sigma(Rh_2)\rightarrow\pi^*(N\text{-}N)$ [4-10, 44]. This band obscures the absorption of low intensity attributed to the $\pi^*(Rh_2)\rightarrow\sigma^*(Rh\text{-}O)$ transition observed at 2.25-2.50 $\mu m^{-1}$ in $[Rh_2(OOCR)_4(H_2O)_2]$ complexes. The energy of the band I strongly depends on the electronic properties of the axial ligands. Nitrogen and sulfur ligands cause a blue shift of the band I. A considerable blue shift of band I in the electronic spectra of **1** and **4** in the presence of L-histidine (Figure 1) indicate that the amino acid molecule is coordinated along the Rh-Rh axis via the nitrogen atom of the imidazole ring. The reaction of L-methionine with these complexes also leads to the formation of adducts with ligand coordinated through sulfur. This follows from the blue shift of the band I and strong increase of absorption in the range 430 – 350 nm (Figure 2).

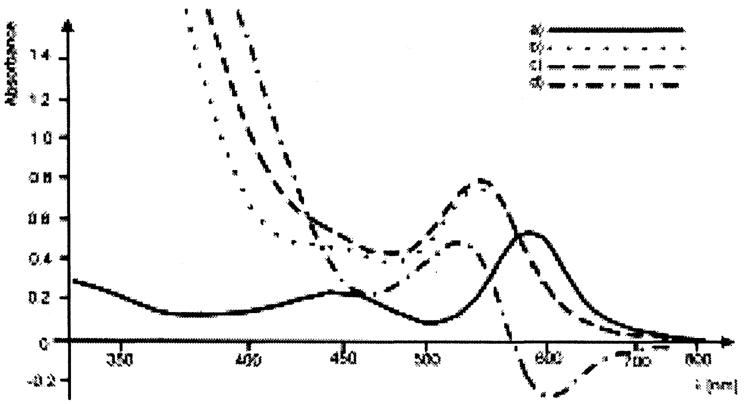

Figure 2. Absorption spectra: a) complex **1**; b) **1**+methionine after 1 min; c) **1**+methionine after 24 hours; d) difference spectrum of (**1**+methionine) / (methionine, **1**) after five days; in 0.01M phosphate buffer, pH=7.4, [**1**]=[$3\cdot10^{-3}$ M] and [histidine]=[$3\cdot10^{-2}$ M]

The latter is attributable to the S → Rh charge transfer [1-10, 44]. This is known that L-methionine forms a stable adduct [45] with rhodium(II) acetate $[Rh_2(OOCCH_3)_4(CH_3SCH_2CH_2CH(NH_3)COO)_2]$. Compounds containing sulfhydryl group, cysteine $HSCH_2CH(NH_2)COOH$, its methyl ester $HSCH_2CH(NH_2)COOMe$ and penicillamine $Me_2C(SH)CH(NH_2)COOH$, in reaction with $[Rh_2(OOCCH_3)_4(H_2O)_2]$ (complex : ligand = 1 : 4) after 2 h gave monomeric paramagnetic rhodium(II) compounds $Rh(L\text{-}H)_2$ (L-H = deprotonated acid) [45].

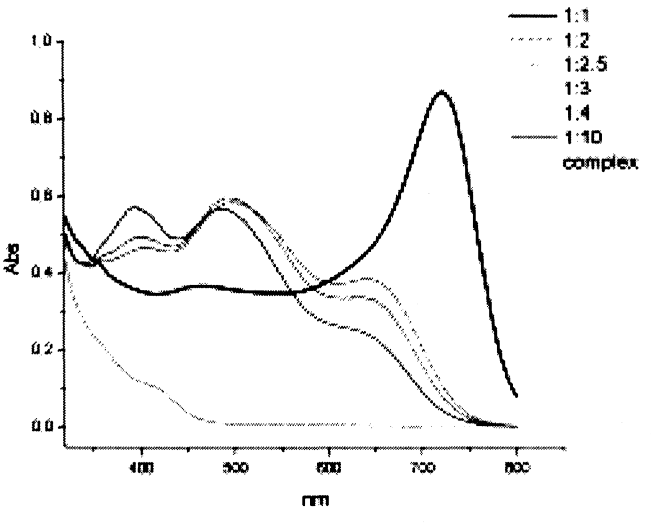

Figure 3. Absorption spectrum of **4** [$2.5\cdot10^{-4}$ M], and spectra of mixtures **4** : GSH at 1:1, 1:2, 1:2.5, 1:3, 1:4 and 1:10 molar ratios. Measured 1 minute after mixing in 0.01M phosphate buffer pH 7.4 under $N_2$.

However, $[Rh_2(\mu\text{-}OOCR)_2(N\text{-}N)_2(H_2O)_2]^{2+}$ complexes (N-N = bpy, phen) in buffer solutions react with compounds containing sulfhydryl group giving rhodium complexes in oxidation states lower than +2. Binuclear rhodium(II) complexes with the $Rh_2^{4+}$ core can be reduced to the Rh(I)-Rh(II) compounds containing $Rh_2^{3+}$ core. There are several examples of such ractions. The $[Rh_2Cl_2(OOCR)_2(N\text{-}N)_2]$ complexes in $CD_3OD$ were reduced at 78 K to $Rh_2^{3+}$ compounds using $^{60}Co$ γ-rays [46] and $[Rh_2(\mu\text{-}OOCR)_2(N\text{-}N)_2(H_2O)_2]^{2+}$ compounds are readily reduced with alcohols giving mixed-valence Rh(I)-Rh(II) molecular wires $[Rh_2(\mu\text{-}OOCR)_2(N\text{-}N)_2]_n(BF_4)_n$ [47]. In the one-electron chemical or electrochemical reduction of $[Rh_2(\mu\text{-}OOCCH_3)_2(bpy)_2(MeCN)_2]^{2+}$ in acetonitrile, the binuclear paramagnetic complex $[Rh_2(\mu\text{-}OOCCH_3)_2(bpy)_2(MeCN)_2]^{+}$ is formed [48]. However, during the reduction of the $[Rh_2(\mu\text{-}OOCR)_2(bpy)_2(H_2O)_2](OOCR)_2$ complexes with hot alcohols, compounds with a $Rh_2^{3+}$ core immediately undergo dimerization giving diamagnetic complex $[Rh_4(\mu\text{-}OOCR)_4(bpy)_4](OOCR)_2$ containing $Rh_4^{6+}$ core [49]. In the electronic spectra of the reduced complexes very intense band at 592 nm and bands of lower intensity at 440 nm, 680 nm and 780 nm were observed [49]. The formation of tetranuclear ($Rh_4^{6+}$), hexanuclear ($Rh_6^{8+}$), and other polynuclear complexes has been observed during the oxidation of complexes with 1,3-diisocyanopropane (dicp) and 2,5-dimethyl-2,5-diisocyanohexane(ddch) $[Rh_2(CNRNC)_4]^{2+}$ (CNRNC = dicp, ddch). Intense bands were observed in the electronic spectra of the polynuclear isocyanide complexes in the region 560 – 900 nm [50-54]. The diamagnetic compound $[Rh_4(\mu\text{-}pz)_4(CNBu^t)_8]$ (pz = pyrazolate) has also been described [55].

Sulfhydryl compounds, GSH, CoA and cysteine, at pH = 7.4 reduce the complex **4** giving compounds which show strong absorption bands in the region 350 – 750 nm. These reactions are very fast; they were followed using stopped flow method. Compound **4** is readily reduced with GSH giving polynuclear rhodium complexes and GSSG. When the **4**:GSH ratio was 1:1, a complex absorbing strongly at 723 nm was formed (Figure 3). This compound is EPR silent. Thus, most probably **4** is reduced to the $Rh_2^{3+}$ complex which immediately dimerizes giving diamagnetic compound with $Rh_4^{6+}$ core. In the next steps, the last compound is reduced to the polynuclear Rh(I) complexes with an absorption at 670 - 350 nm (Figure 3). At **4**:GSH ratios 1:2 - 1:10, the compound with a band at 723 nm is not formed. This indicates that at these **4**:GSH ratios reduction is faster and only polynuclear Rh(I) complexes can be observed (Figure 3).

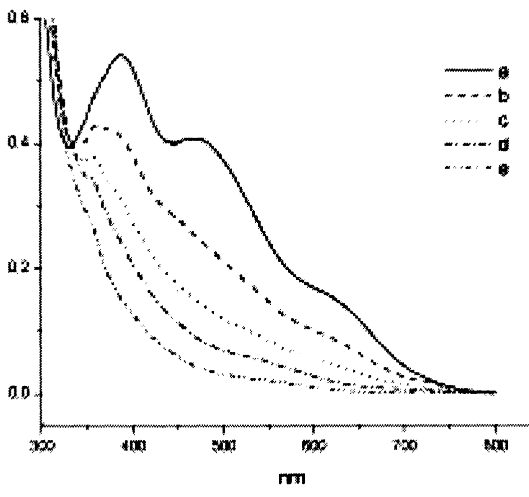

Figure 4. Absorption spectra of complex **4** [$3\cdot10^{-4}$ M] mixed with cysteine [$3\cdot10^{-4}$ M] in 0.01M phosphate buffer pH 7.4 under $N_2$ measured a) 0.1 second, b) 2 minutes, c) 4 minutes, d) 6 minutes and e) 15 minutes after mixing.

Reactions of cysteine and CoA with complex **4** at pH = 7.4 and **4**:Cys(CoA) ratio 1:1 – 1:10 are faster than those with GSH because the bands at 723 nm were not observed, at all Rh:HSR ratios spectra were similar to those formed in the reaction with an excess of GSH (Figure 4 and 5). Complexes **2** and **3** in reactions with GSH give similar products (Figure 6). Complex **1** is not reduced by GSH. In the case of the reaction at pH = 7.4 the band I at 589 nm is only slightly blue shifted and a strong increase of absorption was observed at the wavelength below 500 nm caused by S → Rh CT transition (Figure 7). This indicates that the stable $[Rh_2(O_2CCH_3)_4(GSH)_2]$ adduct is formed. At pH = 4.0 the reduction proceeds easier, complex **4** is reduced with the formation of compounds containing $Rh_4^{6+}$ core, also at greater excess of GSH, and reduction products formed in the reaction with cysteine are stable at least for several hours (Figure 8).

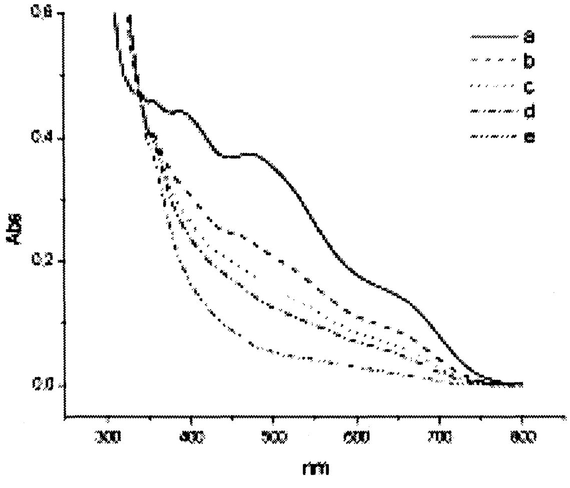

Figure 5. Absorption spectra of complex **4** [$3 \cdot 10^{-4}$ M] mixed with coenzyme A [$3 \cdot 10^{-4}$ M] in 0.01M phosphate buffer pH 7.4 under $N_2$ measured a) 0.1 second, b) 2 minutes, c) 4 minutes, d) 6 minutes and e) 15 minutes after mixing.

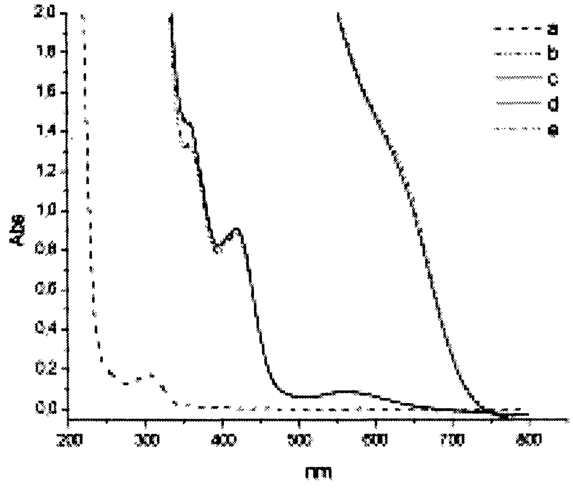

Figure 6. Absorption spectra: a) GSH [$1 \cdot 10^{-3}$ M], b) complex **3** [$5 \cdot 10^{-4}$ M], c) complex **2** [$5 \cdot 10^{-4}$ M] and d) GSH+**3**, e) GSH+**2**, at GSH:complex = 2:1, measured in 0.01M phosphate buffer pH 7.4.

Electronic and CD spectra indicate that complex **4** strongly interact with ceruloplasmin (Figures 9 and 10). The intensity of the "blue" copper band at 610 nm strongly decreased in 5 h after addition of complex **4** to the solution of ceruloplasmin in phosphate buffer (at pH = 7.4). The CD spectra of ceruloplasmin and of a mixture of ceruloplasmin with complex **4** (Figure 10) prove that the addition of compound **4** diminishes considerably the content of α-helix in the protein because of a distinct decrease of intensity of negative transitions at 214 and 221 nm as well as an appearance of the negative transitions at 225 and 228 nm characteristic of β-sheet conformation [56,57]. In CD spectra in the near UV and visible region considerable changes have also been observed (Figure 10). The spectrum of complex **4** show negative transitions at 423, 352 (sh), 307(sh) and 284 nm and positive transitions at 377, 266, 252, 233 and 212 nm. All these transitions correspond to the observed transitions in electronic spectra. However, in the spectrum of complex **4** in the presence of ceruloplasmin the first transition has been shifted to 440nm and second (positive) one to390 nm. However, in this case new transitions have also been found, namely negative peaks at 352 nm and 248 nm and a positive transition at 315 nm. The negative transition at about 281 nm was observed almost at the same energy as in complex **4**.

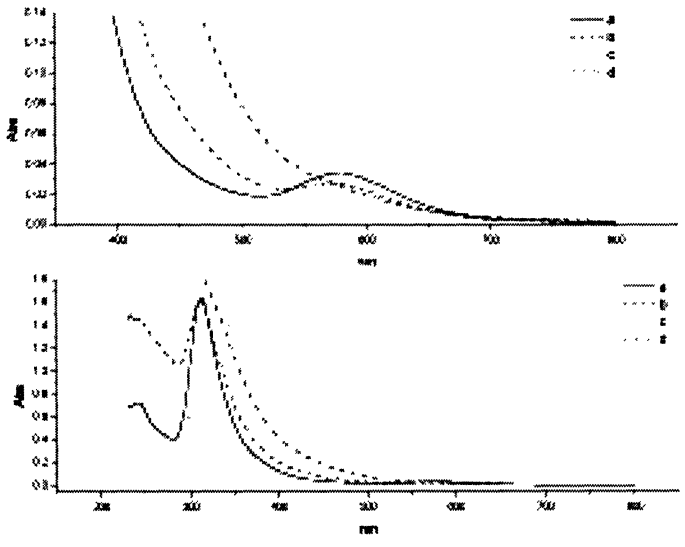

Figure 7. Absorption spectra of complex **1** [$3{\cdot}10^{-4}$ M] mixed with GSH [$3{\cdot}10^{-4}$ M] in 0.01M phosphate buffer pH 7.4 measured a) 0.1 second, b) 1 minute, c) 3 minutes, d) 15 minutes after mixing.

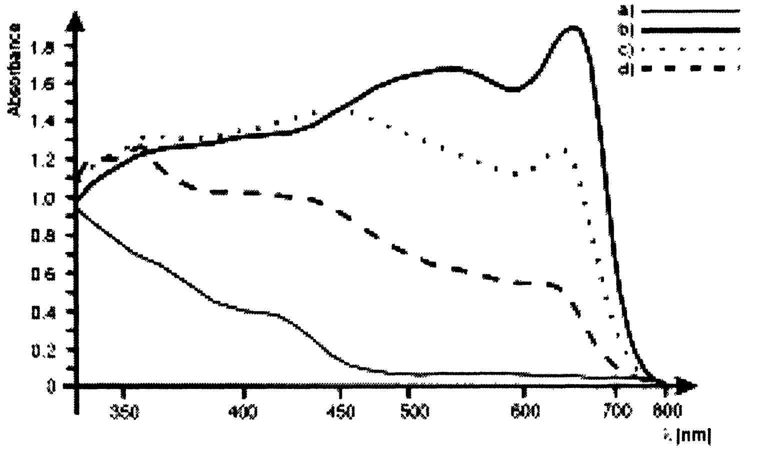

Figure 8. Absorption spectra: a) **4** [$2{\cdot}10^{-4}$ M] and mixture **4**:cysteine at 1:3 molar ratio measured b) 5 minutes, c) 20 minutes, d) 2 hours, after mixing in 0.01M acetate buffer pH 4.0.

These data indicate that $[Rh_2(O_2CR)_2(N\text{-}N)_2(H_2O)_2]^{2+}$ and $[Rh_2Cl_2(O_2CMe)_2(bpy)_2]$ complexes interact with ceruloplasmin changing strongly its structure, considerably decreasing the content of α-helix and decreasing the intensity of the blue copper band at 610 nm. The last effect can be explained by strong changes in the coordination geometry of the two copper atoms of type 1 located in domains 4 and 6 coordinated to two histidines and a cysteine, at a distance of around 2.0 Å and with a methionine at a longer distance of 3.0 Å and most likely change of coordination of copper of type 1 in the domain 2 where methionine was replaced by an alanine at a longer distance of 3.7 Å.

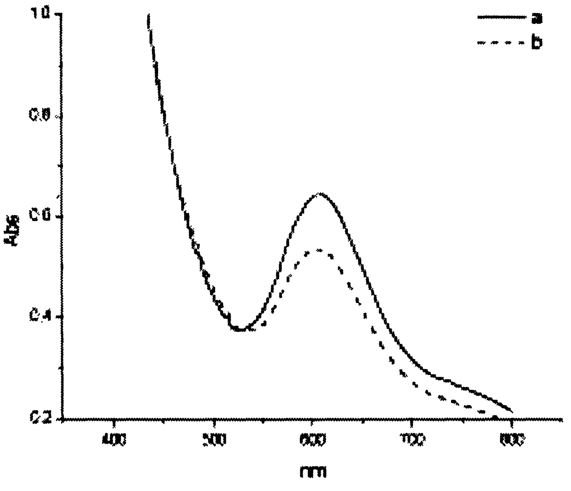

Figure 9. Absorption spectra of complex **4** [$2.5 \cdot 10^{-4}$ M] mixed with ceruloplasmin [$8.9 \cdot 10^{-4}$ M] in 0.01M phosphate buffer pH 7.4 measured a) 0.1second and b) 5 hours after mixing.

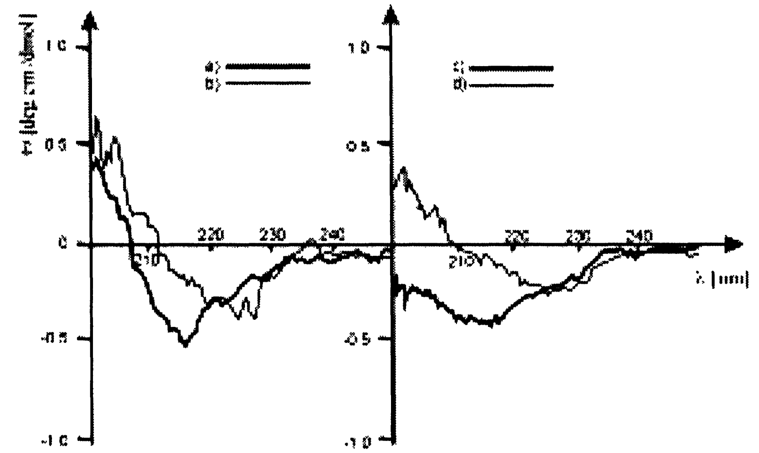

Figure 10. CD spectra: a) ceruloplasmin, b) ceruloplasmin in the presence of **4**, c) apoceruloplasmin and d) apoceruloplasmin in the presence of **4**, measured in 0.01M phosphate buffer pH 7.4

These changes lead to the lost of oxidase activity of ceruloplasmin in the presence of rhodium complexes $[Rh_2(O_2CR)_2(N\text{-}N)_2(H_2O)_2]^{2+}$ and $[Rh_2Cl_2(O_2CMe)_2(bpy)_2]$. The oxidation of *p*-phenylenediamine (PPD) after addition of these complexes to the solution of ceruloplasmin was completely stopped. Ceruloplasmin contains five cysteine-cysteine S-S bridges, three cysteines coordinated with copper and one free cysteine SH group. However, the electronic spectra indicate that complex **4** is not coordinated with sulfhydryl groups or MeS groups of methionines because in the electronic spectra of complex **4** in the presence of ceruloplasmin at different protein : complex **4** ratios, bands characteristic of rhodium complexes containing sulfur ligands were not observed. Thus, most likely, rhodium is coordinated with ceruloplasmin via histidine nitrogen atoms.

**ACKNOWLEDGMENTS**
The authors are grateful to Committee of Scientific Research, KBN, for support of this work (grant No. 3T09A 034 15)

**REFERENCES**

1. F.A. Cotton and R.A. Walton, *Multiple Bonds Between Metal Atoms*, Clarendon Press, Oxford, 1993;
2. E.B. Boyar and S.D. Robinson, *Coord.Chem.Rev.* **50** (1983) 109.

3. T.R. Felthouse, *Progr.Inorg.Chem.* **29** (1982) 73.
4. F. Pruchnik, B.R. James and P. Kvintovics, *Can.J.Chem.* **64** (1986) 936.
5. F. Pruchnik, *Pure Appl.Chem.* **61** (1989) 795.
6. F. Pruchnik, M. Zuber, H. Pasternak and K. Wajda, *Spectrochim. Acta* **34A** (1978) 1111.
7. F.Pruchnik, J. Hanuza, K. Hermanowicz, K. Wajda-Hermanowicz and M. Zuber, *Spectrochim. Acta* **45A** (1989) 835.
8. H. Pasternak, and F. Pruchnik, *Inorg.Nucl.Chem.Letters* **12** (1976) 591.
9. F. Pruchnik and M. Zuber, *Rocz.Chem.* **51** (1977) 1813.
10. T. Glowiak, F. Pruchnik and M. Zuber, *Polish J.Chem.* **65** (1991) 1749.
11. H. Pasternak, E. Lancman and F. Pruchnik, *J.Mol.Catal.* **29** (1985) 13.
12. H. Pasternak, F. Pruchnik, K. Wajda-Hermanowicz and M. Zuber, *Polish J.Chem.* **63** (1989) 619.
13. H. Pasternak and F. Pruchnik, *Polish J.Chem.* **66** (1992) 865.
14. J.L. Bear, H.B. Gray, L. Rainen, I.M. Chang, R. Howard, G. Serio and A.P.Kimball, *Cancer Chemother.Rep.* **59** (1975) 611.
15. R.A. Howard, A.P. Kimball and J.L. Bear, *Cancer Res.* **39** (1979) 2568.
16. K.R. Dunbar, J.H. Matonic, V.P. Saharan, C.A. Crawford and G. Christou, *J.Am.Chem.Soc.* **116** (1994) 2201.
17. R.G. Hughes, J.L. Bear and A.P. Kimball, *Proc.Am.Assoc.Cancer Res.* **13** (1972) 120.
18. P.N. Rao, M.L. Smith, S. Pathak, R.A. Howard and J.L. Bear, *J.Nat.Cancer Inst.* **64** (1980) 905.
19. L.M. Hall, R.J. Speer and H.J. Ridgway, *J.Clin. Hematol. Oncol.* **10** (1980) 25.
20. E. Tselepi-Kalouli and N. Katsaros, *J.Inorg.Biochem.* **40** (1990) 95.
21. L.D. Dale, T.M. Dyson, D.A. Tocher and D.I Edwards, *Anticancer Drug Res.* **4** (1989) 295.
22. B. Esposito, S. Zyngier, A. Souza and R. Najjar, *Metal-Based Drugs* **4** (1997) 333.
23. A Souza, R. Najjar, E. Oliveira and S. Zyngier, *Metal-Based Drugs* **3** (1997) 39.
24. A. Souza, R. Najjar, S. Glikmanas and S. Zyngier, *J.Inorg.Biochem.* **64** (1996) 1.
25. L. Trynda and F.P. Pruchnik, *J.Inorg.Biochem.* **58** (1995) 69.
26. L. Trynda-Lemiesz and F.P. Pruchnik, *J.Inorg.Biochem.* **66** (1997) 187.
27. F.P. Pruchnik and D. Dus, *J.Inorg.Biochem.* **61** (1996) 55.
28. F. Pruchnik and D. Dus, II Symposium on Inorganic Biochemistry and Molecular Biophysics, Proceedings, Wroclaw, 1989, p.281.
29. F.P. Pruchnik, G. Kluczewska, A. Wilczok, U. Mazurek and T. Wilczok, *J.Inorg.Biochem.* **65** (1997) 25.
30. A. Wilczok, Ph.D. Thesis, Silesian Academy of Medicine, Katowice, 1990.
31. F.P. Pruchnik, M. Bien_ and T.M. Lachowicz, *Metal-Based Drugs* **3** (1996) 185
32. M. Bien, T.M. Lachowicz, A. Rybka, F.P. Pruchnik and L. Trynda, *Metal-Based Drugs* **4** (1997) 81.
33. M. Bien, F.P. Pruchnik, A. Seniuk. T.M. Lachowicz and P. Jakimowicz, *J.Inorg.Biochem.* **73** (1999) 49.
34. B. Abolmaali, H.V. Taylor and U. Weser, Struct. Bonding **91** (1998) 91.
35. V.B. Vassiliev, A.M. Kachurin, M. Beltramini, G.P. Rocco, B. Salvato, and V.S. Gaitsboki, *J. Inorg. Biochem.* **65** (1997) 167.
36. F.K. Klemens, J.C. Severns, R. Tamilarasan and D.R. McMillin, *Inorg. Chim. Acta* **250** (1996) 75.
37. I. Zaitseva, V. Zaitsev, G. Card, K. Moshkov, B. Bax, A. Ralph and P. Lindley, *J. Biol. Inorg. Chem.* **1** (1996) 15.
38. J.H. Dawson, D.M. Dooley, R. Clark, P.J. Stephens and H.B. Gray, *J. Am. Chem. Soc.* **101** (1979) 5046.
39. N. Takahashi, T.L. Ortel and F.W. Putman, *Proc. Natl. Acad. Sci. USA* **81** (1984) 390.
40. G.L. Rempel, P. Legzdins, H. Smith and G. Wilkinson, *Inorg. Synth.* **13** (1972) 90.
41. P. Legzdins, R.W. Mitchell, G.L. Rempel, J.D. Ruddick and G. Wilkinson, *J. Chem. Soc.A* (1970) 3322.
42. C.R. Wilson and H. Taube, *Inorg. Chem.* **14** (1975) 405.
43. E.W. Rice, *Clin. Chim. Acta* **5** (1960) 632.
44. L. Natkaniec and F.P. Pruchnik, *J. Chem. Soc. Dalton Trans.* (1994) 3261.
45. G. Pneumatikakis and P. Psaroulis, *Inorg. Chim. Acta* **46** (1980) 97.
46. F.P. Pruchnik, A. Jezierski and E. Kalecinska, *Polyhedron* **10** (1991) 2551.
47. F. P. Pruchnik, P. Jakimowicz, Z. Ciunik, K. Stanislawek, L. A. Oro, C. Tejel, M. A. Ciriano, submitted for publication.
48. C.A. Crawford, J.H. Matonic, J.C. Huffman, K. Folting, K.R. Dunbar and G. Christou, *Inorg. Chem.* **36** (1997) 2361.
49. E. Galdecka, Z. Galdecki, F.P. Pruchnik and P. Jakimowicz, *Trans. Met. Chem.*, **25** (2000), 315.
50. C. Tejel, M. A. Ciriano, J. A. Lopez, F. A. Lahoz, L.A. Oro, *Angew. Chem. Int. Ed.*, **37** (1998), 1542; C. Tejel, M. A. Ciriano, L.A. Oro, *Chem Eur. J.*, **5** (1999), 1131, and references therein.
51. V.M. Miskowski, I.S. Sigal, K.R. Mann, H.B. Gray, S.J. Milder, G.S. Hammond and P.R. Ryason, *J. Am. Chem. Soc.* **101** (1979) 4383.
52. V.M. Miskowski, G.L. Nobinger, D.S. Kliger, G.S. Hammond, N.S. Lewis, K.R. Mann and H.B. Gray, *J. Am. Chem. Soc.* **100** (1978) 485.
53. I.S. Sigal, K.R. Mann and H.B. Gray, *J. Am. Chem. Soc.* **102** (1980) 7252.
54. I.S. Sigal and H.B. Gray, *J. Am. Chem. Soc.* **103** (1981) 2220.
55. N.S. Lewis, K.R. Mann, J.G. Gordon II and H.B. Gray, *J. Am. Chem. Soc.* **98** (1976) 7461.
56. S.F. Mason, *Molecular Optical Activity and the Chiral Discrimation*, Cambridge University Press, Cambridge, 1982.

57. K. Nakanishi, N. Berova and R.W. Woody, (Ed.), *Circular Dichroism. Principles and Applications*, VCH, Weinheim, 1994.

**Received: June 26, 2000 - Accepted: September 5, 2000 -**
**Received in revised camera-ready format: October 6, 2000**

